# Acid Sphingomyelinase Promotes Cellular Internalization of *Clostridium perfringens* Iota-Toxin

**DOI:** 10.3390/toxins10050209

**Published:** 2018-05-20

**Authors:** Masahiro Nagahama, Masaya Takehara, Kazuaki Miyamoto, Kazumi Ishidoh, Keiko Kobayashi

**Affiliations:** 1 Department of Microbiology, Faculty of Pharmaceutical Sciences, Tokushima Bunri University, Yamashiro-cho, Tokushima 770-8514, Japan; mtakehara@ph.bunri-u.ac.jp (M.T.); stnqxka5192@mc.pikara.ne.jp (K.M.); kobakei@ph.bunri-u.ac.jp (K.K.); 2 Division of Molecular Biology, Institute for Health Sciences, Tokushima Bunri University, Yamashiro-cho, Tokushima 770-8514, Japan; kishidoh@tokushima.bunri-u.ac.jp

**Keywords:** *C. perfringens* iota-toxin, membrane repair, acid sphingomyelinase

## Abstract

*Clostridium perfringens* iota-toxin is a binary actin-ADP-ribosylating toxin composed of the enzymatic component Ia and receptor binding component Ib. Ib binds to a cell surface receptor, forms Ib oligomer in lipid rafts, and associates with Ia. The Ia-Ib complex then internalizes by endocytosis. Here, we showed that acid sphingomyelinase (ASMase) facilitates the cellular uptake of iota-toxin. Inhibitions of ASMase and lysosomal exocytosis by respective blockers depressed cell rounding induced by iota-toxin. The cytotoxicity of the toxin increased in the presence of Ca^2+^ in extracellular fluids. Ib entered target cells in the presence but not the absence of Ca^2+^. Ib induced the extracellular release of ASMase in the presence of Ca^2+^. ASMase siRNA prevented the cell rounding induced by iota-toxin. Furthermore, treatment of the cells with Ib resulted in the production of ceramide in cytoplasmic vesicles. These observations showed that ASMase promotes the internalization of iota-toxin into target cells.

## 1. Introduction

*Clostridium perfringens* iota-toxin belongs to a binary actin-ADP-ribosylating toxin family featuring two individual proteins implicated in enterotoxemia among young domestic livestock [1,2,3,4,5,6]. One component of iota-toxin, Ia, ADP-ribosylates monomeric actin, interferes with the actin polymerization within a host cell and then disintegrates its cytoskeletal organization [6,7,8]. The other component, Ib, binds to the lipolysis-stimulated lipoprotein receptor (LSR) on the cell surface and accelerates cellular uptake of Ia into a host cell [6,9]. Each component alone lacks cytotoxicity. Members of the binary actin-ADP-ribosylating family are *Clostridium botulinum* C2 toxin, *Clostridium difficile* toxin (CDT), *Clostridium spiroforme* toxin (CST), and *Bacillus cereus* vegetative insecticidal protein (VIP) [1,2,3,4].

Iota-toxin is endocytosed into target cells and causes cytotoxicity by taking advantage of intracellular transport [3,4,5,6]. Ib recognizes LSR on the target cell membrane, forms heptameric transmembrane pores in lipid-rafts of plasma membrane, and associates with Ia [3,4,5,6,7]. The Ia-Ib complex is then incorporated into the cells [3,4,5,6]. We previously reported the intracellular trafficking of Ib [6,10,11,12]. Ib is endocytosed, trafficked to early endosomes, and delivered to recycling endosomes and late endosomes. Then, Ib is sorted from late endosomes to lysosomes for breakdown [6,12]. We also demonstrated that Ib alone caused cytotoxicity to A431 and A549 cells [13]. Ib induced the rapid depletion of intracellular ATP and cell swelling in both cells. Moreover, an ultrastructural study confirmed the necrotic cell death of Ib-treated cells. These results indicated that Ib causes cell necrosis of sensitive cells [13].

Many pathogenic bacteria, including *Neisseria gonorrhoeae*, *N. meningitidis*, *Listeria monocytogenes*, *Pseudomonas aeruginosa*, and *Escherichia coli*, invade host cells by utilizing the acid sphingomyelinase (ASMase) and ceramide (Cer) [14,15,16,17,18]. ASMase and Cer are responsible for the internalization of pathogenic bacteria and the induction of various types of host cell damage [14,15,16,17,18]. Pore-forming toxins (PFTs) are important cytolytic factors liberated by bacterial pathogens that protect bacteria against the host innate immune defenses, impair epidermal barriers, and release substances essential for persistent bacterial proliferation. PFT plasma membrane pores result in Ca^2+^ influx. PFT-induced Ca^2+^ influx promotes lysosomal exocytosis and lysosomal ASMase release into the extracellular space [15,16]. Subsequently, ASMase induces endocytosis, which serves to repair membrane damage [19,20,21].

We recently reported that *C. botulinum* C2 toxin needs ASMase for cell entry [22]. Ca^2+^ influx induced by C2 toxin facilitates the exocytosis of lysosomal ASMase. The extracellularly released ASMase cleaves sphingomyelin in the outer membrane, which results in the production of ceramide-rich microdomains. The microdomains bud into cytoplasmic membranes, initiating endocytosis [6]. It is unclear whether ASMase is involved in the internalization of iota-toxin in host cells. Madin–Darby canine kidney (MDCK) cells offer an excellent model for studying the uptake of iota-toxin [12]. In the present study, we examined whether ASMase participates in the endocytosis of iota-toxin.

## 2. Results

### 2.1. Calcium Ion Accelerates Endocytosis of Iota-Toxin

**Figure 1:**
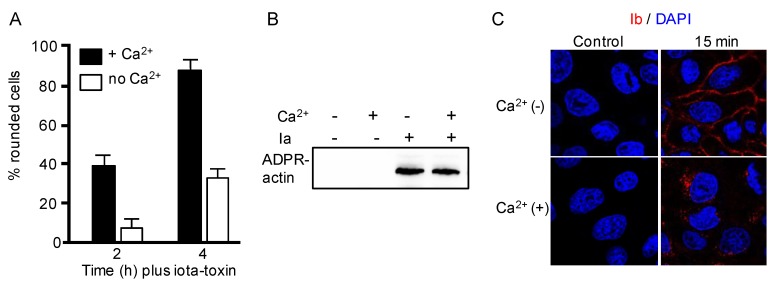
Role of Ca^2+^ on action of iota-toxin. (**A**) Madin–Darby canine kidney (MDCK) cells were treated either in Ca^2+^ (1.8 mM) media or Ca^2+^-free media with Ia (200 ng/mL) and Ib (400 ng/mL) at 37 °C for the indicated incubation periods. Photographs of the cells were taken. The number of rounded cells and total number of cells were counted from the photographs, and the number of round cells was indicated as a percentage of the values for the untreated control (means ± standard deviation (SD) (*n* = 3)); (**B**) Detection of ADP-ribosylated actin (ADPR-actin). Ia was incubated with biotin-labeled NAD+ and beta-actin in the presence or absence of Ca^2+^ (1.8 mM) buffer at 37 °C for 60 min. Biotin-ADP-ribosylated actin was analyzed by western blotting. Representative data from one of three studies are shown; (**C**) MDCK cells were treated with Ib (1 μg/mL) at 37 °C for 15 min. Cells were fixed, permeabilized, and stained with an anti-Ib antibody and 4′,6′-diamino-2-phenylindole (DAPI). Ib (red) and the nucleus (blue) were viewed using a confocal microscope. The same studies were performed four times, and a typical result is shown. Bar, 7.5 μm.

We investigated the role of Ca^2+^ in target cell entry of iota-toxin. Having previously demonstrated that the cell rounding activity of iota-toxin was enhanced by increased extracellular Ca^2+^ concentrations (0.2–1.8 mM) [23], we evaluated the cytotoxicity of iota-toxin in the absence or presence of Ca^2+^. MDCK cells treated with iota-toxin in extracellular Ca^2+^ medium caused quick cell rounding (Figure 1A). In contrast, in calcium-free extracellular medium, the toxin evoked weak cell rounding. An in vitro ADP-ribosylation assay demonstrated that iota-toxin was able to ADP-ribosylate the G-actin both in the absence and presence of Ca^2+^ (Figure 1B). To examine the role of Ca^2+^ on the entry of Ib, MDCK cells were treated with Ib in the absence or presence of Ca^2+^ at 37 °C (Figure 1C). Ib effectively entered the cytoplasm in the presence of Ca^2+^. In contrast, in medium that lacked Ca^2+^, Ib was present on the cell membrane. We previously showed that the binding and oligomerization of Ib to MDCK cells was detected in Ca^2+^-free buffer, as well as in Ca^2+^-containing buffer [23], indicating that the binding and oligomer formation of Ib to target cells do not need extracellular Ca^2+^. Pore-forming toxin-induced Ca^2+^ influx initiates endocytosis during membrane repair [20,21]. We already reported that Ib causes Ca^2+^ influx into MDCK cells [12]. These results indicate that Ib facilitates Ca^2+^ uptake from the extracellular fluid during endocytic internalization.

### 2.2. Ib Promotes Exocytosis of Lysosomal Acid Sphingomyelinase

**Figure 2:**
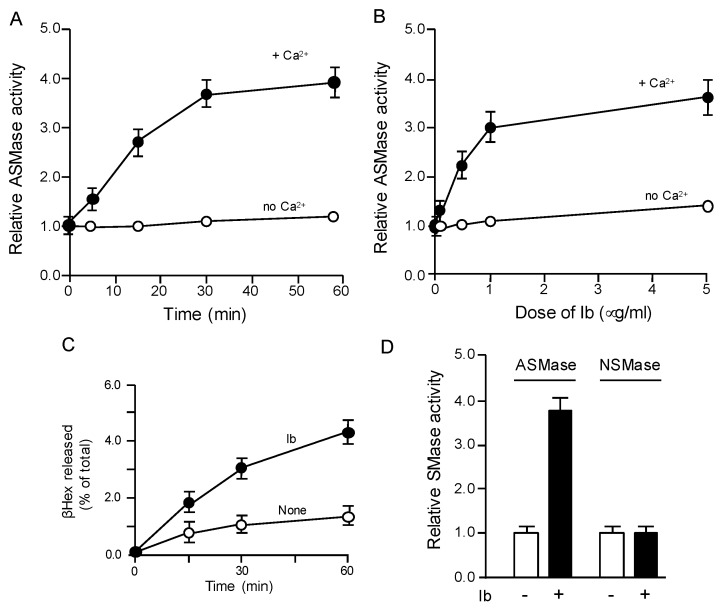
Iota-toxin induces release of acid sphingomyelinase. (**A**) MDCK cells were treated either in Ca^2+^ (1.8 mM) medium or Ca^2+^-free medium with Ib (400 ng/mL) at 37 °C for the indicated incubation periods; (**B**) MDCK cells were treated either in Ca^2+^ (1.8 mM) medium or Ca^2+^-free medium with various amounts of Ib at 37 °C for 30 min. Acid sphingomyelinase (ASMase) activity in the culture medium was measured as described in the Materials and Methods. Non-treated cells employed as controls are set at a basal level of 1.0. Results are indicated as relative values of the values acquired from non-treated controls (means ± standard deviations (SD) (*n* = 4)); (**C**) MDCK cells were treated with Ib (500 ng/mL) at 37 °C for the indicated incubation periods. Supernatant fluids from the culture were evaluated for *β*-hexosaminidase (*β*Hex) activity. *β*Hex activities are represented as the percentage of the total enzymatic activity detected in supernatant fluid and cells (means ± standard deviations (SD) (*n* = 4)); (**D**) Cells were treated with Ib (400 ng/mL) for 60 min at 37 °C. ASMase activity in supernatant fluids from the culture and neutral sphingomyelinase (NSMase) activity in cell were evaluated as described in the Materials and Methods. Results are expressed as percentages of the values acquired from non-treated controls (means ± standard deviations (SD) (*n* = 4)).

Ca^2+^-triggered exocytosis of the lysosomal enzyme ASMase promotes endocytosis of lesions [19,20,21]. As depicted in Figure 2A,B, Ib elevates ASMase activity in the extracellular medium in a concentration- and time-dependent fashion in Ca^2+^ medium, but not in Ca^2+^-free medium. The origin of ASMase in the extracellular medium is proposed to be lysosomal exocytosis. To examine whether Ib causes the exocytosis of lysosomes, the extracellular medium from the cells treated with Ib was evaluated for the presence of *β*-hexosaminidase (*β*Hex) as a lysosomal marker protein [22] (Figure 2C). *β*Hex activity was time-dependently elevated in the extracellular medium from the Ib-incubated cells compared to the control cells. This result indicated that Ib facilitates lysosomal exocytosis. We next examined whether Ib induces the activation of neutral sphingomyelinase (NSMase) (Figure 2D). The extracellular medium from Ib-treated cells exhibited increased ASMase activity. In contrast, Ib did not induce an increase in NSMase activity.

### 2.3. Role of Acid Sphingomyelinase on Cellular Uptake of Iota-Toxin

**Figure 3:**
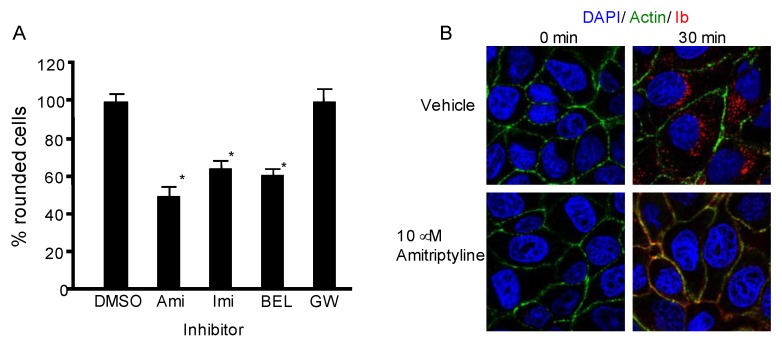
Inhibition of acid sphingomyelinase blocks iota-toxin-caused cytotoxic activity. (**A**) MDCK cells were preincubated with various inhibitors (25 μM) at 37 °C for 1 h. The cells were treated with Ia (200 ng/mL) and Ib (400 ng/mL) at 37 °C for 4 h. The number of rounded cells and total number of cells were counted from the photographs, and the number of round cells is shown as a percentage of the values for the untreated control (means ± standard deviation (SD) (*n* = 4)). Significant differences (Student’s *t*-test) from control cells are shown. * *p* < 0.01, significantly different from DMSO plus iota-toxin. Abbreviations: DMSO, dimethyl sulfoxide; Ami, amitriptyline; Imi, imipramine; BEL, bromoenol lactone; (**B**) MDCK cells were preincubated with amitriptyline (10 μM) at 37 °C for 1 h. Cells were incubated with Ib (1 μg/mL) at 37 °C for 30 min. Cells were fixed, permeabilized, and stained with the anti-Ib antibody, Alexa Fluor488-phallodin and DAPI. Ib (red), actin (green), and the nucleus (blue) were analyzed using a confocal microscope. A representative data from one of three studies is shown. Bar, 7.5 μm.

We investigated whether ASMase is involved in the cellular internalization of iota-toxin. MDCK cells were treated with ASMase inhibitors amitriptyline and imipramine and evaluated for susceptibility to iota-toxin (Figure 3A). The cell rounding activity caused by iota-toxin was suppressed by treatments with amitriptyline (Ami) and imipramine (Imi). Bromoenol lactone (BEL), an inhibitor of lysosomal exocytosis, also inhibited the toxin-induced cytotoxicity. In contrast, GW4869 (GW), which is a specific inhibitor of NSMase, did not influence the cell rounding caused by the toxin. Then, we examined the effect of amitriptyline on cellular uptake of Ib (Figure 3B). Ib was localized in intracellular vesicles in the presence of a vehicle. In contrast, incubation of Ib with MDCK cells that were preincubated with amitriptyline decreased the Ib internalization, and Ib was instead observed in the cell membrane. These results reveal that ASMase is involved in Ib cell entry into target cells.

### 2.4. Effects of ASMase-siRNA on Iota-Toxin-Caused Cytotoxicity

**Figure 4:**
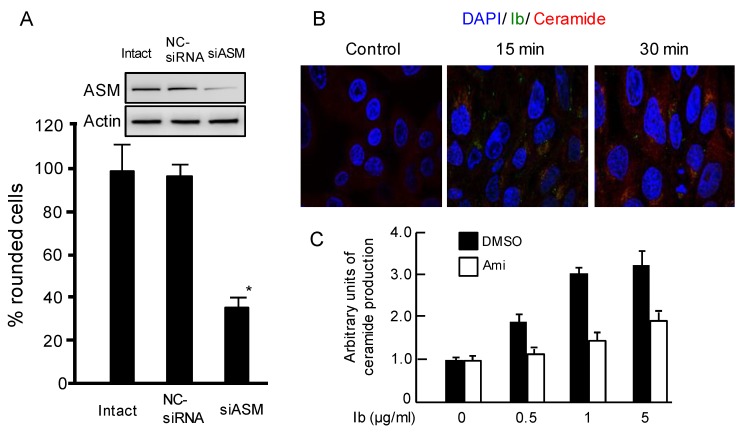
Role of acid sphingomyelinase on iota-toxin-induced cytotoxic effects. (**A**) The expression of ASMase in MDCK cells transfected with siASM was analyzed by western blotting using antibodies against ASMase and beta-actin. Representative data from one of three experiments are shown. Ia (200 ng/mL) and Ib (400 ng/mL) were incubated with intact cells, negative control (NC)-siRNA-transfected cells, or siASMase-transfected cells at 37 °C for 4 h. The number of rounded cells and total number of cells were counted from the photographs, and the number of round cells is shown as a percentage of the values for the untreated control (means ± standard deviation (SD) (*n* = 3)); (**B**) MDCK cells were treated with Ib (500 ng/mL) at 37 °C for 15 and 30 min, washed, fixed, and permeabilized. The cell nuclei were stained with DAPI (blue). Ceramide was stained with an anti-ceramide antibody and observed using a confocal microscope. These results are typical data of three experiments. Bar, 7.5 μm. (**C**) MDCK cells were pretreated with DMSO or amitriptyline (Ami, 25 μM) at 37 °C for 1 h. The cells were incubated with Ib at 37 °C for 30 min. Ceramide productions was detected as depicted in Materials and Methods. The level of ceramide was determined by densitometry. The level of control cells was set to 1. Values represent means ± S.D. (*n* = 3).

To examine the influence of ASMase on iota-toxin-caused cell rounding activity, an RNAi analysis was used to silence ASMase. Treating cells with ASMase siRNA resulted in decreased expression of ASMase as compared to intact cells or negative control (NC)-siRNA-transfected cells (Figure 4A). Compared with intact or NC-siRNA, ASMase siRNA transfection could markedly reduce iota-toxin-induced cell rounding (Figure 4A). These results showed that ASMase plays a role in iota-toxin-induced cytotoxicity.

### 2.5. Iota-Toxin Causes An Increase in Ceramide

To confirm the role of ASMase in the toxin-caused cytotoxicity, we investigated whether Ib causes the production of ceramide in target cells. Subcellular localization of ceramide production caused by Ib was visualized by immunofluorescence staining using an anti-ceramide antibody. As shown in Figure 4B, Ib caused an elevation of ceramide production in a time-dependent fashion. Ib colocalized with ceramide in intracellular vesicles after 30 min, indicating that the generation of ceramide in the outer leaflet of the plasma membrane by Ib promotes Ib entry. Then, we examined whether Ib causes the production of ceramide. When MDCK cells were incubated with Ib at 37 °C for 30 min, Ib induced the production of ceramide in a dose-dependent fashion (Figure 4C). Ib-induced ceramide production was inhibited by the addition of amitriptyline. Following heat denaturation, inactivated Ib did not cause the ceramide production.

## 3. Discussion

In this study, Ib facilitated ASMase release and then ceramide production in the exterior cell surface. Formation of ceramide platforms in the plasma membrane induces a negative curvature in the membrane, leading to an inward vesiculation for its endocytic uptake. The present results indicated that ASMase plays a critical role in the entry of iota-toxin into target cells.

Pore formation of PFT in the cell membrane causes the host cell injury. Nucleated cells repair plasma membrane wounding through a molecular mechanism underlying Ca^2+^-dependent lysosomal exocytosis, ASMase release, and rapid endocytosis of lesion sites. Some bacteria gain access to target cells by producing PFTs and SMases. In a previous study, Ib formed cation-selective channels in lipid bilayer membranes [4]. We reported that Ib causes K^+^ release from Vero cells [10] and shows cytotoxicity in A549 cells [13]. Moreover, Ib facilitates Ca^2+^ influx during cell entry [12]. In this research, Ib was readily endocytosed under conditions of Ca^2+^ buffer but not Ca^2+^-free buffer. We speculate that extracellular release of ASMase by Ca^2+^-mediated lysosomal exocytosis is involved in an early stage of Ib entry to the host cells. In this experiment, Ib induced the extracellular release of lysosomal ASMase. The cytotoxic activity of iota-toxin toward target cells was inhibited by ASMase inhibitors and ASMase knockdown. Moreover, Ib increased the production of ceramide. It has been reported that Ca^2+^ influx in injured cells stimulates a repair pathway that leads to resealing of the cell membrane [24]. Ca^2+^-induced exocytosis of lysosomes facilitates the membrane repair of host cells wounded by PFTs [25,26]. Exocytosis of lysosomes in injured cells is associated with an increased endocytosis that eliminates pores formed by PFTs and lesion sites from the cell membrane [26]. Ca^2+^-induced exocytosis of the lysosomal ASMase accelerates the endocytosis that facilitates active membrane repair pathway in injured cells. ASMase hydrolyses the cell outer membrane sphingomyelin into ceramide [27], and newly generated ceramide-rich platforms can cause inward bending of the affected plasma membrane [28,29]. Therefore, Ib utilizes this plasma membrane repair mechanism as an entry pathway. These studies indicate that iota-toxin is incorporated into host cells through Ca^2+^-triggered lysosomal ASMase exocytosis.

Iota-toxin-caused cytotoxicity was not blocked by NSMase inhibitor GW4869. Additionally, NSMase was not activated by Ib treatment. According to these findings, NSMase does not play a role in the cytotoxicity of iota-toxin. In the case of iota-toxin, hydrolysis of sphingomyelin by ASMase delivered by Ib produces a negative curvature in the outer layer. This negative curvature leads to an inward vesiculation for endocytosis. ASMase is responsible for cellular uptake of iota-toxin, while NSMase is involved in the generation of ceramide-riched microdomain in the inner layer of the plasma membrane, thereby leading to an outward curvature that is implicated in the exosomal shedding of PFTs [30]. Ib treatment of MDCK cells cannot induce cell blebbing relating to the exosomal shedding. Again, NSMase has no involvement in the cytotoxicity of iota-toxin.

Sphingomyelin is abundant in the cytoplasmic membrane and is observed in the cholesterol- and sphingolipid-rich plasma membrane domains, termed lipid rafts [21]. Enzymatic hydrolysis of sphingomyelin by exocytosed ASMase produces ceramide. Ceramide supports lipid raft enlargement into large membrane domains in which cell-surface receptors cluster. LSR is a single-pass transmembrane receptor expressed in various tissues including the liver and intestine [31]. LSR serves as the cellular receptor for clostridial binary toxins, namely iota-toxin, *C. difficile* CDT, and *C. spiroforme* toxin [9,32]. It has been reported that the binding component of CDT causes accumulation of LSR into lipid rafts [31]. Previously, we reported that Ib binds to a host cell receptor, accumulates in plasma membrane lipid rafts, and internalizes into the cells [11]. Our findings indicate that Ib induces the clustering of LSR into lipid rafts and is endocytosed from ceramid-rich lipid rafts.

## 4. Conclusions

We demonstrated an important function of ASMase in cellular uptake of iota-toxin. Ib-induced elevation of intracellular Ca^2+^ concentration promotes lysosomal exocytosis. An extracellularly secreted ASMase generates the invaginated ceramide microdomain into the cell, triggering endocytosis that leads to the cellular uptake of iota-toxin.

## 5. Materials and Methods

### 5.1. Materials

Recombinant Ia and Ib were purified as described earlier [10]. The Anti-Ib antibody was obtained from rabbits immunized with purified Ib [13]. Amitriptyline hydrochloride and imipramine hydrochloride were purchased from Fujifilm Wako Pure Chem (Osaka, Japan). GW4869 hydrate, bromoenol lactone (BEL), monoclonal anti-ceramide IgM monoclonal antibody (clone: 15B4) produced in mouse, beta-actin and *p*-nitrophenyl *N*-acetyl-*β*-d-glucosaminide were purchased from Merck (Tokyo, Japan). Rabbit anti-3-*β*-actin antibody and anti-acid sphingomyelinase (H-181) antibody were purchased from Santa Cruz Biotechnology (Santa Cruz, CA, USA). Amplex Red Sphingomyelinase Assay Kit, Hanks’ balanced salt solution (HBSS), Dulbecco’s modified Eagle’s medium (DMEM), Alexa Fluor 488 phalloidin conjugate, 4′,6′-diamino-2-phenylindole (DAPI), Alexa Fluor 568-conjugated goat anti-rabbit IgG, Alexa Fluor 488-conjugated goat anti-rabbit IgG, and Alexa Flour 564-conjugated goat anti-mouse IgM were purchased from Thermo Fisher Sci. (Tokyo, Japan). Enhanced chemiluminescence (ECL, Saint Paul, MI, USA) kits, peroxidase-conjugated streptavidin, and horseradish peroxidase-labeled anti-rabbit IgG were obtained from GE Healthcare (Tokyo, Japan).

### 5.2. Cell Culture Assay

MDCK (Madin Darby Canine Kidney) cells were purchased from the Riken BRC (Tsukuba, Japan). Cells were cultivated in DMEM containing 10% fetal bovine serum (FBS), 100 μg/mL of streptomycin, 100 units/mL of penicillin, and 2 mM L-glutamine (FBS-DMEM). Cells were incubated at 37 °C in a 5% CO_2_ atmospheres under humidified conditions. For cytotoxicity experiments, cells were seeded in 48-well tissue culture plates and incubated in FCS-DMEM. Various concentrations of Ia and Ib were added and cells were further incubated at 37 °C. After 4 h, cells were observed for morphological changes as described earlier [13]. For evaluation of the role of Ca^2+^ during iota-toxin-induced cytotoxicity, culture media were removed and replaced with HBSS with or without 1.8 mM Ca^2+^. For inhibitor treatment, cells were incubated for 30 min with 25 μM imipramine, 25 μM amitriptyline, 25 μM GW4869, or 25 μM BEL at 37 °C. Thereafter, iota-toxin was added and cells were additionally incubated at 37 °C in the presence of toxin plus inhibitor. In some experiments, an inactive form of Ib was produced by heating for 5 min at 95 °C. For detection of ASMase and beta-actin, Western blot analysis was performed using specific antibodies as described earlier [22].

### 5.3. Measurement of Acidic or Neutral Sphingomyelinase Activities

After incubation with iota-toxin, the culture media of the treated cells were treated with neutral lysing buffer (20 mM Tris-HCl buffer, pH 7.5, 1% Triton X-100, 1 mM EDTA) or were treated with acidic lysing buffer (50 mM sodium acetate buffer, pH 5.0, 1% Triton X-100, 1 mM EDTA) and newly-added protease inhibitor mixture (Nacalai tesque Inc, Kyoto, Japan). Resultant enzyme activities of ASMase (at pH 5.0) and neutral sphingomyelinase (NSMase) (at pH 7.5) were assayed utilizing an Amplex Red Sphingomyelinase Assay Kit as described earlier [22].

### 5.4. Assay of *β*-Hexosaminidase

MDCK cells were seeded in 24-well plates and incubated with Ib or PBS at 37 °C. After the given incubation periods, the culture supernatants were removed, and centrifuged at 10,000 *g* for 20 min. Activity of *β*-hexosaminidase in the supernatants was determined by incubation with 1 mM *p*-nitrophenyl *N*-acetyl-*β*-d-glucosaminide in 0.1 M citrate buffer (PH 4.5) at 37 °C for 2 h. To stop the reaction, 0.1 M sodium carbonate buffer (pH 9.8) was added to the reaction mixture. Absorbance at 405 nm was measured. Activity of *β*-hexosaminidase was indicated as a percentage of the total enzyme activity detected in the culture medium and cell lysate generated using 1% Triton X-100 as described earlier [22].

### 5.5. Assay of ADP-Ribosylation

Five micrograms of beta-actin were incubated at 37 °C for 60 min in a reaction buffer solution containing 25 mM Tris-HCl buffer (pH 7.6), 2.5 mM MgCl_2_, 0.5 mM DTT, 0.5 mM EDTA, protease inhibitor mixture, 5 µM biotin-labelled NAD^+^ (Trevigen Inc., Gaithersburg, MD, USA) and 200 ng of Ia protein. The reaction was terminated by addition of 2× SDS-sample buffer and heating at 95 °C for 5 min. The samples were subjected to SDS-PAGE and blotted to a PVDF membrane. The biotin-labelled ADP-ribosytated actin was determined using peroxidase-conjugated streptavidin and a subsequent ECL kit [8].

### 5.6. Si RNA Transfection

siRNA negative controls and siRNAs for SMPD1 were purchased from Qiagen (Tokyo, Japan). MDCK cells (5 × 10^6^ cells) were incubated with 500 pmol siRNA and then transfected using a Neon^TM^ transfection system (Thermo Fisher Sci, Tokyo, Japan) [22]. After electroporation, cells were seeded to 24-well plates and incubated at 37 °C. Experiments were performed at 48 h after siRNA transfection [22].

### 5.7. Immunocytochemistry

MDCK cells were seeded on a poly-d-lysine-coated glass bottom dish (MatTek, Ashland, MA, USA) and cultured in a 5% CO_2_ at 37 °C atmosphere for 24 h in FCS-DMEM. To examine the cellular uptake of Ib, cells were treated with Ib at 37 °C for the given incubation periods in FCS-DMEM. Cells were washed three times with phosphate-buffered saline (PBS) and treated with 2% paraformaldehyde at room temperature. The bottom dishes were incubated for 15 min in 20 mM NH_4_Cl, and incubated in PBS supplemented with 0.1% Triton X-100 at room temperature for 15 min. Cells were blocked in PBS supplemented with 4% BSA at room temperature for 2 h. For Ib staining, cells were incubated with primary antibody (rabbit anti-Ib antibody) at 4 °C for 12 h. The cells were then rinsed with PBS containing 0.02% Triton X-100, and incubated with secondary antibody (Alexa Fluor 488-conjugated anti-rabbit IgG or Alexa Fluor 568-conjugated anti-rabbit IgG or anti-mouse IgG-FITC) at room temperature for 1 h. Nuclei were labeled with DNA dye DAPI (0.4 μg/mL). For ceramide staining, cells were treated with primary antibody (mouse anti-ceramide IgM antibody) for 2 h, and with secondary antibody (anti-mouse IgM Alexa Fluor594) for 1 h. Cells were analyzed using a Nikon A1 laser scanning confocal microscope (Tokyo, Japan) as described earlier [12,22].

### 5.8. Determination of Ceramide 

MDCK cells (1 x 10^7^ cells/mL) were incubated with Ib for 30 min at 37 °C. Then, the ceramide production was stopped by supplementing chloroform/methanol/H_2_O (4:4:1) solvent mixture, and ceramide was extracted. Ceramide was phosphorylated by 1,2-diacylglycerol kinase (Merck, Tokyo, Japan) using [γ-^32^P]-ATP (Perkin Elmer, Tokyo, Japan) and analyzed by thin-layer chromatography (TLC). The TLC was monitored by autoradiographic method [22].

### 5.9. Statistical Analysis

Statistical analyses were carried out by EZR software (Easy R, Saitama Medical Center, Jichi Medical Univeristy, Japan) [33]. Differences between the two groups were assessed by a two-tailed Student’s *t-*test. A one-way analysis of variance (ANOVA) followed by the Tukey test were utilized to check for differences among three or more groups. *P* values < 0.01 were considered statistically significant.

## References

[1] Sakurai J., Nagahama M., Oda M., Tsuge H., Kobayashi K. (2009). *Clostridium perfringens* iota-toxins: Structure and function. Toxins (Basel).

[2] Aktories K., Lang A.E., Schwan C., Mannherz H.G. (2011). Actin as target for modification by bacterial protein toxins. FEBS J..

[3] Aktories K., Schwan C., Papatheodorou P., Lang A.E. (2012). Bidirectional attack on the actin cytoskeleton. Bacterial protein toxins causing polymerization or depolymerization of actin. Toxicon.

[4] Stiles B.G., Pradhan K., Fleming J.M., Samy R.P., Barth H., Popoff M.R. (2014). *Clostridium* and *Bacillus* binary enterotoxins: Bad for the bowels, and eukaryotic being. Toxins (Basel).

[5] Knapp O., Benz R., Popoff M.R. (2015). Pore-forming activity of clostridial binary toxins. Biochim. Biophys. Acta.

[6] Takehara M., Takagishi T., Seike S., Oda M., Sakaguchi Y., Hisatsune J., Ochi S., Kobayashi K., Nagahama M. (2017). Cellular entry of *Clostridium perfringens* iota-toxin and *Clostridium botulinum* C2 toxin. Toxins (Basel).

[7] Tsuge H., Nagahama M., Oda M., Iwamoto S., Utsunomiya H., Marquez V.E., Katunuma N., Nishizawa M., Sakurai J. (2008). Structural basis of actin recognition and arginine ADP-ribosylation by *Clostridium perfringens* iota-toxin. Proc. Natl. Acad. Sci. USA.

[8] Tsurumura T., Tsumori Y., Qiu H., Oda M., Sakurai J., Nagahama M., Tsuge H. (2013). Arginine ADP-ribosylation mechanism based on structural snapshots of iota-toxin and actin complex. Proc. Natl. Acad. Sci. USA.

[9] Papatheodorou P., Carette J.E., Bell G.W., Schwan C., Guttenberg D., Brummelkamp T.R., Aktories K. (2011). Lipolysis-stimulated lipoprotein receptor (LSR) is the host receptor for the binary toxin *Clostridium difficile* transferase (CDT). Proc. Natl. Acad. Sci. USA.

[10] Nagahama M., Nagayasu K., Kobayashi K., Sakurai J. (2002). Binding component of *Clostridium perfringens* iota-toxin induces endocytosis in Vero cells. Infect. Immun..

[11] Nagahama M., Yamaguchi A., Hagiyama T., Ohkubo N., Kobayashi K., Sakurai J. (2004). Binding and internalization of *Clostridium perfringens* iota-toxin in lipid rafts. Infect. Immun..

[12] Nagahama M., Umezaki M., Tashiro R., Oda M., Kobayashi K., Shibutani M., Takagishi T., Ishidoh K., Fukuda M., Sakurai J. (2012). Intracellular trafficking of *Clostridium perfringens* iota-toxin b. Infect. Immun..

[13] Nagahama M., Umezaki M., Oda M., Kobayashi K., Tone S., Suda T., Ishidoh K., Sakurai J. (2011). *Clostridium perfringens* iota-toxin b induces rapid cell necrosis. Infect. Immun..

[14] Grassmé H., Gulbins E., Brenner B., Ferlinz K., Sandhoff K., Harzer K., Lang F., Meyer T.F. (1997). Acidic sphingomyelinase mediates entry of *N. gonorrhoeae* into nonphagocytic cells. Cell.

[15] Utermöhlen O., Karow U., Lohler J., Krönke M. (2003). Severe impairment in early host defense against *Listeria monocytogenes* in mice deficient in acid sphingomyelinase. J. Immunol..

[16] Grassmé H., Jendrossek V., Riehle A., von Kurthy G., Berger J., Schwarz H., Weller M., Kolesnick R., Gulbins E. (2003). Host defense against *Pseudomonas aeruginosa* requires ceramide-rich membrane rafts. Nat. Med..

[17] Falcone S., Perrotta C., De Palma C., Pisconti A., Sciorati C., Capobianco A., Rovere-Querini P., Manfredi A.A., Clementi E. (2004). Activation of acid sphingomyelinase and its inhibition by the nitric oxide/cyclic guanosine 3′,5′-monophosphate pathway: Key events in *Escherichia coli*-elicited apoptosis of dendritic cells. J. Immunol..

[18] Simonis A., Hebling S., Gulbins E., Schneider-Schaulies S., Schubert-Unkmeir A. (2014). Differential activation of acid sphingomyelinase and ceramide release determines invasiveness of *Neisseria meningitidis* into brain endothelial cells. PLoS Pathog..

[19] Tam C., Idone V., Devlin C., Fernandes M.C., Flannery A., He X., Schuchman E., Tabas I., Andrews N.W. (2010). Exocytosis of acid sphingomyelinase by wounded cells promotes endocytosis and plasma membrane repair. J. Cell Biol..

[20] Los F.C.O., Randis T.M., Aroian R.V., Ratner A.J. (2013). Role of pore-forming toxins in bacterial infectious diseases. Microbiol. Mol. Biol. Rev..

[21] Andrews N.W., Almeida P.E., Corrotte M. (2014). Damage control: Cellular mechanisms of plasma membrane repair. Trends Cell. Biol..

[22] Nagahama M., Takehara M., Takagishi T., Seike S., Miyamoto K., Kobayashi K. (2017). Cellular uptake of *Clostridium botulinum* C2 Toxin requires acid sphingomyelinase activity. Infect. Immun..

[23] Kobayashi K., Nagahama M., Ohkubo N., Kojima T., Shirai H., Iwamoto S., Oda M., Sakurai J. (2008). Role of Ca^2+^-binding motif in cytotoxicity induced by *Clostridium perfringens* iota-toxin. Microb. Pathog..

[24] McNeil P.L., Miyake K., Vogel S.S. (2003). The endomembrane requirement for cell surface repair. Proc. Natl. Acad. Sci. USA.

[25] Walev I., Bhakdi S.C., Hofmann F., Djonder N., Valeva A., Aktories K., Bhakdi S. (2001). Delivery of proteins into living cells by reversible membrane permeabilization with streptolysin-O. Proc. Natl. Acad. Sci. USA.

[26] Idone V., Tam C., Goss J.W., Toomre D., Pypaert M., Andrews N.W. (2008). Repair of injured plasma membrane by rapid Ca^2+^-dependent endocytosis. J. Cell Biol..

[27] Schissel S.L., Schuchman E.H., Williams K.J., Tabas I. (1996). Zn^2+^-stimulated sphingomyelinase is secreted by many cell types and is a product of the acid sphingomyelinase gene. J. Biol. Chem..

[28] Holopainen J.M., Angelova M.I., Kinnunen P.K. (2000). Vectorial budding of vesicles by asymmetrical enzymatic formation of ceramide in giant liposomes. Biophys. J..

[29] Trajkovic K., Hsu C., Chiantia S., Rajendran L., Wenzel D., Wieland F., Schwille P., Brügger B., Simons M. (2008). Ceramide triggers budding of exosome vesicles into multivesicular endosomes. Science.

[30] Draeger A., Babiychuk E.B. (2013). Ceramide in plasma membrane repair. Handb. Exp. Pharmacol..

[31] Papatheodorou P., Hornuss D., Nölke T., Hemmasi S., Castonguay J., Picchianti M., Aktories K. (2013). *Clostridium difficile* binary toxin CDT induces clustering of the lipolysis-stimulated lipoprotein receptor into lipid rafts. MBio.

[32] Papatheodorou P., Wilczek C., Nölke T., Guttenberg G., Hornuss D., Schwan C., Aktories K. (2012). Identification of the cellular receptor of *Clostridium spiroforme* toxin. Infect. Immun..

[33] Kanda Y. (2013). Investigation of the freely available easy-to-use software ‘EZR’ for medical statistics. Bone Marrow Transplant..

